# Elucidating metabolic pathways through genomic analysis in highly heavy metal-resistant *Halobacterium salinarum* strains

**DOI:** 10.1016/j.heliyon.2024.e40822

**Published:** 2024-11-30

**Authors:** Houda Baati, Mariem Siala, Souad Benali, Chafai Azri, Christopher Dunlap, Rosa María Martínez-Espinosa, Mohamed Trigui

**Affiliations:** a Research Laboratory of Environmental Sciences and Sustainable Development, LR18ES32, University of Sfax, Tunisia; b United States Department of Agriculture, National Center for Agricultural Utilization Research, Crop Bioprotection Research Unit, 1815 North University St, Peoria, IL, 61604, USA; c Biochemistry and Molecular Biology and Edaphology and Agricultural Chemistry Department, Faculty of Sciences, University of Alicante, Ap. 99, E-03080, Alicante, Spain; d Applied Biochemistry Research Group, Multidisciplinary Institute for Environmental Studies “Ramón Margalef”, University of Alicante, Ap. 99, E-03080, Alicante, Spain

**Keywords:** Halophilic archaea, *Halobacterium salinarum*, Comparative genomics, Metabolism, Biotechnological applications

## Abstract

The annotated and predicted genomes of five archaeal strains (AS1, AS2, AS8, AS11 and AS19), isolated from Sfax solar saltern sediments (Tunisia) and affiliated with *Halobacterium salinarum*, were performed by RAST webserver (Rapid Annotation using Subsystem Technology) and NCBI prokaryotic genome annotation pipeline (PGAP). The results showed the ability of strains to use a reduced semi-phosphorylative Entner-Doudoroff pathway for glucose degradation and an Embden-Meyerhof one for gluconeogenesis. They could use glucose, fructose, glycerol, and acetate as sole source of carbon and energy. ATP synthase, various cytochromes and aerobic respiration proteins were encoded. All strains showed fermentation capability through the arginine deiminase pathway and facultative anaerobic respiration using electron acceptors (Dimethyl sulfoxide and trimethylamine N-oxide). Several biosynthesis pathways for many amino acids were identified. Comparative and pangenome analyses between the strains and the well-studied halophilic archaea *Halobacterium* NRC-1 highlighted a notable dissimilarity. Besides, the strains shared a core genome of 1973 genes and an accessory genome of 767 genes. 129, 94, 67, 15 and 29 unique genes were detected in the AS1, AS2, AS8, AS11 and AS19 genomes, respectively. Most of these unique genes code for hypothetical proteins. The strains displayed plant-growth promoting characteristics under heavy metal stress (Ammonium assimilation, phosphate solubilization, chemotaxis, cell motility and production of indole acetic acid, siderophore and phenazine). Therefore, they could be used as a biofertilizer to promote plant growth. The genomes encoded numerous biotechnologically relevant genes responsible for vitamin biosynthesis, including cobalamin, folate, biotin, pantothenate, riboflavin, thiamine, menaquinone, nicotinate, and nicotinamide. The carotenogenetic pathway of the studied strains was also predicted. Consequently, the findings of this study contribute to a better understanding of the halophilic archaea metabolism providing valuable insights into their ecophysiology as well as relevant biotechnological applications.

## Highlights

• The strains shared a core genome of 1973 genes and an accessory genome of 767 genes.
• A reduced semi-phosphorylative Entner-Doudoroff pathway was revealed.
• Several biosynthesis pathways for many amino acids were identified.
• Genes involved in plant growth promotion and vitamins biosynthesis were identified.

## Graphical abstract

**Figure:**
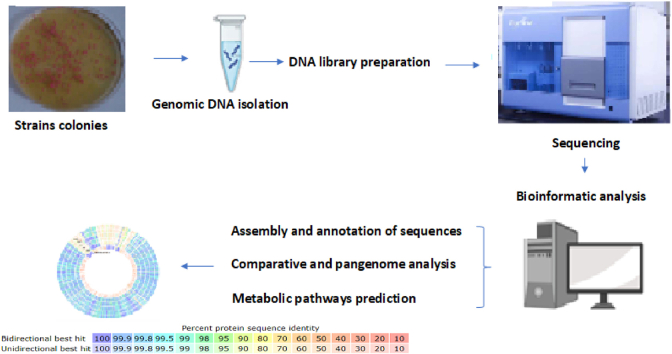

## 1 Introduction

Hypersaline environments are poly-extreme ecosystems that are not only characterized by high salinity level, but also by other extreme environmental parameters (temperature, osmotic pressure and radiation) [1]. Extremely halophilic archaea, abundant microorganisms present in these environments, are well adapted to the extreme conditions with an optimal growth occurring at 3.5–4.5 M NaCl [2]. They develop various molecular adaptation mechanisms for protecting cells against abiotic stressors; syntheses of stress proteins, DNA repair mechanisms and biosynthesis of secondary metabolites [[1], [2], [3], [4], [5]]. They have attracted great scientific interest due to their extraordinary molecular adaptations to the high salinity conditions of the ecosystems where they live. Several studies focused on haloarchaeal biomolecules: proteins, lipids, enzymes and secondary metabolites [6]. Genomic approaches have been developed for various extremophilic microorganisms, improving the understanding of adaptation to harsh conditions at the molecular level [[7], [8], [9], [10], [11], [12], [13], [14]].

Studies on halophilic archaeal genomes are still scarce compared to bacterial genomes [2,5,15]. Various recent studies on genomic analysis of many halophilic archaeal genera (*Natrinema*, *Halobacterium*, *Halomicroarcula*, *Halorutilus*, *Halosegnis*, *Halonotius*) are addressed to reveal their mechanisms and osmoadaptative strategies to cope with extreme environmental conditions [1,[4], [5], [6],^8^,[15], [16], [17], [18], [19], [20], [21]]. However, only few recent studies are focused on major metabolic pathways used to grow under hypersaline environments such as central carbohydrate metabolism (glycolysis/gluconeogenesis, pyruvate metabolism and tricarboxylic acid cycle), respiratory chain, pentose phosphate pathway and amino acids biosynthesis [[22], [23], [24], [25], [26]].

The halophilic archaea metabolism is not uniform due to their different nutritional requirements, carbon sources, and metabolic pathways exhibiting considerable diversity [[27], [28], [29], [30], [31]]. Most of such archaea are aerobic heterotrophs, growing with amino acids and/or carbohydrates as sources of carbon and energy [2]. They obtain necessary nutrients from other microorganisms inhabiting their environments [32]. Due to their ability for facultative anaerobic growth, the halophilic archaea can use a variety of specific pathways. These varied considerably depending on different electron acceptors namely nitrate, nitrite, per(chlorate), or dimethylsulfoxide [[33], [34], [35]].

The genus *Halobacterium,* belonging to the *Halobacteriaceae* family, is an extremely halophilic archaea that is widespread in salted food and worldwide hypersaline environments, including saline lakes, salterns, halite, ancient salt deposits and saline soils [6,[36], [37], [38]]. This genus has been widely studied as a model for archaeal extremophiles [39,40]. It plays a crucial role in the ecology of hypersaline environments and the biogeochemical cycles [41]. Additionally, *Halobacterium* is known for its ability to survive under various stress conditions, not limited to salinity, but also desiccation, UV radiation, and heavy metals exposure [[42], [43], [44]]. Members of this species are aerobic heterotrophs and exhibit complex nutritional demands [18]. *Halobacterium salinarum* is characterized by its versatile physiology permitting its good growth, even under extreme conditions. It regulates accurately metabolic pathways in response to carbon source availability [6,37,45,46].

*Halobacterium salinarum* isolated from highly contaminated Sfax solar saltern superficial sediments (22 strains) were already tested for their ability to resist to selected heavy metals (Cd, Pb, Ni, Zn and Cu) by using agar dilution methods and growth kinetics. The results revealed that they were tolerant to high concentrations of Pb, Ni and Cd up to 2.5–4.5 mM [47,48]. The genomic analysis of five selected strains (AS1, AS2, AS8, AS11 and AS19) showed that they protect cell homeostasis and avoid heavy metal toxicity by reducing influx/enhanced efflux through three classes of transmembrane transporters (P-type ATPase, CDF and ABC transporters), enzymatic detoxification, transcription regulators, and secondary metabolites (carotenoid, exopolysaccharide and siderophores). The highest heavy metal strain (named AS1) showed significant heavy metal tolerance. Compared to the other strains, it was shown the most resistant to heavy metals and may be used in the bioremediation of multi-metal contaminated environments [4]. The potential biotechnological applications of the studied strains were evaluated in terms of hydrolytic enzyme activities and carotenoids production. The results revealed that they produce several hydrolytic enzymes of potential interest in biotechnology as well as carotenoids with significant antioxidant activities [49].

In this study, we have carried out a comparative genomic analysis of the five strains (AS1, AS2, AS8, AS11, and AS19) with the well-studied halophilic archaea *Halobacterium* NRC-1. In addition, we have performed an in-depth investigation of their major metabolic pathways, focusing on central carbohydrate metabolism (glycolysis/gluconeogenesis and tricarboxylic acid cycle), respiratory chains, pentose phosphate pathway and amino acids metabolism, to understand how these microorganisms can grow under hypersaline environments. The results here reported, shed light on metabolic capabilities of these strains that could be of interest for biotechnological applications and design of circular economy based proceses.

## 2 Materials and methods

### 2.1 Site description

**Fig. 1:**
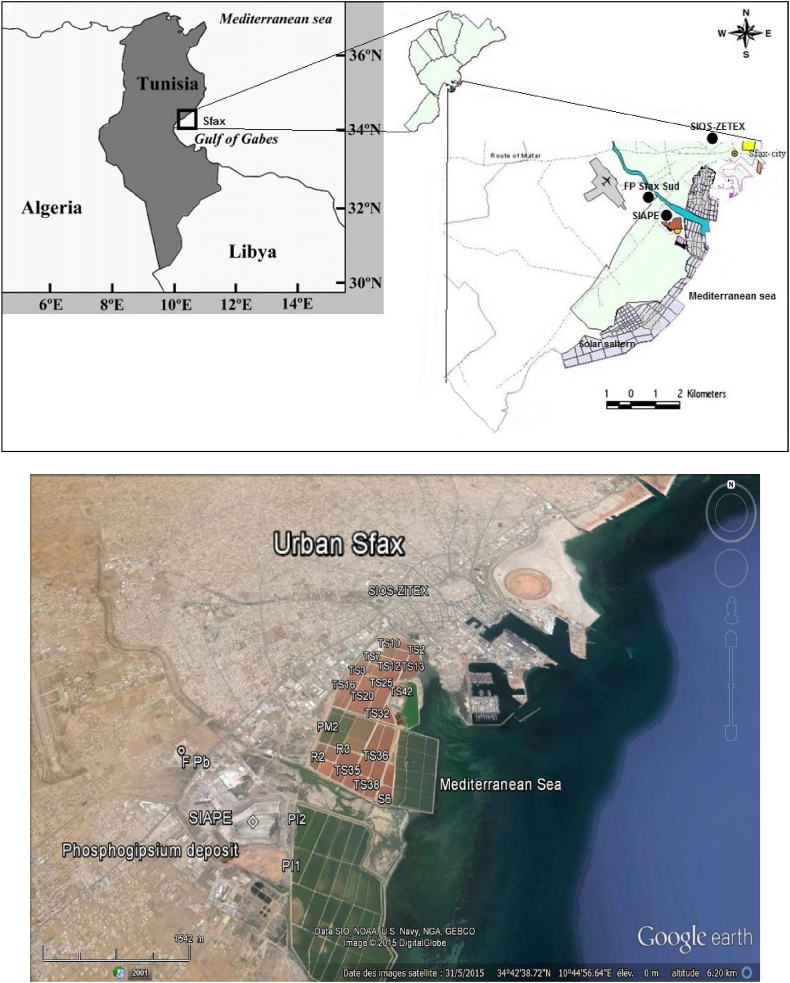
Location map of the solar saltern of Sfax (Tunisia) and sampling sites.

The Sfax solar saltern (Tunisia) is located in the vicinity of the coastline not far from the main industrial pole of the city (34°39 N-10°42 E; Fig. 1). The saltern salinity varies from 37 to 400 g/L [50]. It consists of a series of shallow evaporation ponds (20–70 cm deep), connected by a series of canals and pipelines. These ponds were designed to produce NaCl crystals through the evaporation of seawater. As evaporation progresses, brines are directed into ponds possessing progressively higher salt concentrations. This process continues until the dissolved salts reach saturation and precipitate out of the solution. Since 1950s, this solar saltern is fully subject to the influence of industrial fallouts highly enriched with heavy metals [47,[50], [51], [52]]. The concentration ranges of selected heavy metals (Ni, Cd, Pb, Cu and Zn) are grouped in Table S1.

### 2.2 Archaeal strains, growth maintenance and identification

The strains (named AS1, AS2, AS8, AS11 and AS19) were already isolated from Sfax solar saltern sediment samples in May 2017 [47]. The growth medium was DSC-97. It contained (per litre): yeast extract, 10 g; casamino-acid, 7.5 g; NaCl, 250 g; MgSO_4_.7H_2_O, 20 g; KCl, 2 g; and trisodium citrate, 3 g. The strains were identified by 16S ribosomal RNA gene sequencing. Phylogenetic analysis indicated that the strains were affiliated with *Halobacterium salinarum* [49].

### 2.3 Biochemical tests

Biochemical activities and organic substrates utilization as sole sources of carbon and energy were evaluated using API 20E kits (bioMérieux), according to the instructions provided by manufacturers, using a synthetic medium containing (g/L): NaCl, 250; MgSO_4_·7H_2_O, 20 and KCl, 2 (pH 7). The API 20E detection system is constituted of several tubes containing dehydrated substrates that can be inoculated with a log-phase bacterial suspension [53]. The ability of the strains to use other carbon sources was also tested in the synthetic medium supplemented with fructose, glycerol and maltose (each at 10 g/L).

### 2.4 Genome sequencing and assembly

The five strains were cultivated for 10 days, in DSC-97 medium at 37 °C and 110 rpm. The obtained cells were harvested by centrifugation (8000 g for 30 min), and the genomic DNA of each strain was extracted using Genomic DNA Purification Kit (NucleoSpin Tissue Kit, macherey Nagel) [4]. Genomic DNA of the strains was prepared for sequencing using the Nextera XT library preparation kit (Illumina Inc). The libraries were sequenced using a MiSeq V3 (2 x 300 bp) kit at the National Center for Agricultural Utilization Research, in Peoria, IL, USA. The sequences were quality-trimmed and assembled using CLC Genomics Workbench 20.0 (Qiagen Inc). The genomes were checked for completeness and contamination using CheckM [54].

### 2.5 Gene prediction and annotation

The genes in the assembled sequences were then predicted and annotated automatically using the RAST webserver (Rapid Annotation using Subsystem Technology) and the NCBI prokaryotic genome annotation pipeline (PGAP) [55,56]. The KEGG (Kyoto Encyclopedia of Genes and Genomes) database (http://www.genome.jp/kegg/) was used to identify metabolic pathways [57]. General features of the studied genomes and their accessions numbers deposited at DDBJ/ENA/GenBank are given in Table 1 [4].

**Table 1:** Main features of the sequenced genomes of the five archaeal strains.

Genomic feature	Strain				
	AS1	AS2	AS8	AS11	AS19
**Size (bp)**	2,060,688	2,467,461	2,236,624	2,432,692	2,428,727
**Coverage (%)**	202	109	158	551	217
**G + C content (%)**	65.5	66.0	67.0	66.2	66.2
**Contigs**	558	195	79	48	67
**r + tRNA**	46	49	50	50	50
**Subsystems**	159	170	167	168	168
**Coding sequence**	2803	2836	2476	2695	2710
**Hypothetical sequences**	1315	1305	1082	1500	1423
**Accessions number**	JAHCLW000000000	JAHTKV000000000	JAHTKW000000000	JAHTKX000000000	JAHTKY000000000

### 2.6 Comparative and pangenome analysis

The RAST SEED viewer sequenced-based comparison tool [58] was used for comparative genomic analysis of the five archaeal strains with *Halobacterium* NRC-1 used as a reference sequence. A threshold of 80 % sequence identity was used. The tool colours each gene based on protein similarity in blue (highest gene sequence similarity) or red (absence of homologs) in comparison with the reference genome. The pangenome analysis consisting of three different groups known as the core (conserved genes for all strains), accessory (genes shared by more than two strains), and unique (strain-specific genes) genomes was also carried out [17]. A threshold of 80 % sequence identity was used to identify sequence highly conserved. Since AS2 genome is the largest, it was selected to be the reference strain (Table S2).

### 2.7 Growth rate experiments

The five archaeal strains were grown in a defined media similar (in composition) to DSC-97 without peptone and yeast extract (0.5 g/L), with glucose, fructose, glycerol and acetate as separate carbon sources at a concentration of 10 g/L. The pH was adjusted to 7 by using NaOH (1 N). 1 mL of the mid-log culture of each strain was inoculated in 100 mL of each media in a 250 mL Erlenmeyer flask. All the flasks were incubated at 37 °C and 110 rpm in a shaking incubator (Daihan Lab Tech CO, LTD) for 10 days. The culture growth was monitored at 24-h intervals at 600 nm by UV–Vis spectrophotometer (Genesys 10 SUV-Vis). The experiment was repeated three times to ensure reproducibility. Data related to DO_600_ obtained from triplicate analysis have been subjected to treatment of mean, standard deviation and *t*-test at P < 0.05, to minimize probable errors related to experimental measurement and accuracy in data analysis. Analysis of variance (t-tests) could be used to estimate the probability that the underlying phenomena are truly different. In the current study, differences have been deemed significant at P < 0.05. An average value of DO_600_ of each culture has been used for further interpretation because the reproducibility was at 95 % confidence level. The results have shown good accuracy, with recovery rates for DO_600_ values between 97.2 and 100.3 for analyzed cultures. The mean values and standard deviation (±SD) of the three replicates were calculated using Microsoft Excel 2016. The growth rate of the studied strains was calculated as described previously by Baati et al. [49].

## 3 Results and discussion

### 3.1 Phenotypic and physiological characteristics of the strains

As described previously [49], the five archaeal strains were Gram-negative, motile, and rod-shaped. The catalase and oxidase tests were positive. The strains were not able to grow in media without NaCl. They can grow at 20–35 % (w/v) as total concentration of salts and their optimal growth was observed at 25 % (w/v) salts. They were able to grow at pH 5–9 (optimum pH 7, except for the strain AS1 which has an optimum pH of 6). Concerning the temperature, the strains grew from 25 to 45 °C and optimally at 37 °C.

Additional biochemical activities (in the present work) using the API 20E and culture tests showed that the five strains have the same profile. They were negative for β-galactosidase, arginine dihydrolase, lysine decarboxylase, ornithine decarboxylase, urease, indole, H_2_S and acetoin production. Cells were only positive for tryptophan deaminase and nitrate reductase. The strains were able to grow using differents sources of carbon such as sugar (D-Glucose, D-Rhamnose, D-Saccharose, D-Melibiose, L-Arabinose, D-Fructose and D-Maltose), alcohol (D-Mannitol, inositol, D-Sorbitol and glycerol) and organic acid (Citrate).

### 3.2 Comparative and pangenome analyses

**Fig. 2:**
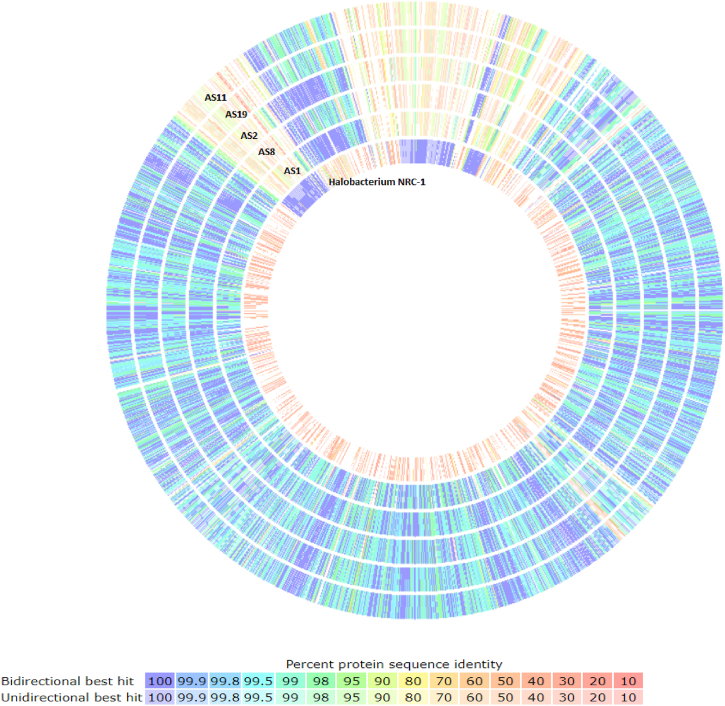
Comparative genome analysis of the five archaeal strains (AS1, AS2, AS8, AS11 and AS19) using the RAST SEED viewer with *Halobacterium* NRC-1 as reference. A threshold of 80 % sequence identity was used. The blue lines represent the highest genes sequence similarity and the red lines indicate the absence of homologs.

The phylogenetic analysis based on 16S rRNA sequencing data suggested that the strains are closely related to *Halobacterium* NRC-1. The taxonomy of the strains was confirmed using average nucleotide identity sharing greater than 98.9 % nucleotide identity with *Halobacterium* NRC-1 [4]. Here, a comparative and a pangenome analysis of the studied archaeal genomes with their closely related species *Halobacterium* NRC-1, already available in the databases, were carried out in the RAST server. Fig. 2 illustrates the results of the comparative analysis highlighting both the variables and conserved regions within the studied genomes. The AS1 and AS2 genomes revealed a high similarity between them. Since AS2 strain has the largest genome, it was selected as the reference strain for comparison. Table S2 lists the gene lengths and their identity score (in percentage). High sequence identity was observed between all five studied strains, and notable dissimilarities were evident when compared to *Halobacterium* NRC-1. Using a sequence identity threshold of 80 %, pangenome analysis identified a core genome consisting of 1973 genes and an accessory genome consisting of 767 genes. The core genome consists of genes involved in energy, carbohydrate, amino acid, coenzyme, lipid and nucleotide metabolism, translation, regulation, ribosomal biogenesis, replication, recombination, repair proteins and those associated with osmotic stress and heavy metal resistance. Whereas the accessory genome consists of genes related to transcription regulations (AsnC, HxlR, AcrR, PadR and HxlR families), mobile genetic elements (Transposases), enzymes (Phosphatase and sulfatase) and some genes responsible for metal transport/resistance (Copper-translocating P-type ATPases, Copper(I) chaperone “CopZ”) and osmotic stress adaptation (Heat shock protein G homolog) were also identified. Genes encoding hypothetical proteins, whose biological roles have not yet been experimentally characterized, were identified in 39.78 % (785) of the core genome and 71 % (545) of accessory genomes. Pangenome analysis revealed that AS1, AS2, AS8, AS11 and AS19 contain 129, 94, 67, 15 and 29 unique genes in their genomes, respectively. Most of these unique genes code for hypothetical proteins. Recently, a comparative genomic analysis on *Halobacterium salinarum* strains isolated from other ecological niches (Salted foods and animal hides) revealed a core genome of 1101 genes [6].

Although the five studied strains are affiliated to *Halobacterium* NRC-1 by 16S rRNA genes sequencing (99.7–100 %) and by ANI values calculation (>95 %), comparative genome analyzes revealed that they are different from each other and from the reference strain *Halobacterium* NRC-1.

### 3.3 Metabolic pathways

#### 3.3.1 Glycolysis/gluconeogenesis

Glycolysis which is responsible for sugars metabolism, particularly glucose, consists of a series of consecutive reactions converting glucose into pyruvate with ATP production [59]. Upon analysis of the five archaeal strains genomes using the KEGG database and RAST analysis, all the genes coding for enzymes of the Embden-Meyerhof (EM) glycolytic pathway were found, except for the enzyme phosphofructokinase. These enzymes include hexokinase, glucose-6-phosphate isomerase, fructose-bisphosphate aldolase, triosephosphate isomerase, glyceraldehyde-3-phosphate dehydrogenase, phosphoglycerate kinase, phosphoglycerate mutase, enolase and pyruvate kinase (Table 2).

**Table 2:** Genes involved in glycolysis/gluconeogenesis, pyruvate metabolism, tricarboxylic acid cycle and respiratory chain.

Genes names	Strain				
	AS1	AS2	AS8	AS11	AS19
**Glycolysis** (**Embden-Meyerhof pathway)/gluconeogenesis**					
- Hexokinase (EC 2.7.1.1)	1	1	1	1	1
- Glucose-6-phosphate isomerase (EC 5.3.1.9)	1	1	1	1	1
- Fructose-bisphosphate aldolase, archaeal class I (EC 4.1.2.13)	1	1	1	1	1
- Triosephosphate isomerase (EC 5.3.1.1)	1	1	1	1	1
- Glyceraldehyde-3-phosphate dehydrogenase (EC 1.2.1.9)	1	1	1	1	1
- Phosphoglycerate kinase (EC 2.7.2.3)	1	1	1	1	1
- Phosphoglycerate mutase (EC 5.4.2.12)	2	1	1	1	1
- Enolase (EC 4.2.1.11)	2	1	1	1	1
- Pyruvate kinase (EC 2.7.1.40)	1	1	1	1	1
- Phosphoenolpyruvate synthase (EC 2.7.9.2)	1	1	1	1	1
- Fructose-1,6-bisphosphatase, type I (EC 3.1.3.11)	1	1	1	1	1
**Glycolysis (Entner-Douderoff pathway)**					
- Glucose 1-dehydrogenase (EC 1.1.1.47)	1	1	1	1	1
- 2-dehydro-3-deoxyphosphogluconate aldolase (EC 4.1.2.14)	5	4	4	4	4
**Pyruvate metabolism and tricarboxylic acid cycle**					
- 2-oxoglutarate/2-oxoacid ferredoxin oxidoreductase, β,γ subunit (EC 1.2.7)	2	1	1	1	1
- Biotin carboxylase of acetyl-CoA carboxylase (EC 6.3.4.14)	2	2	2	2	2
- Dihydrolipoamide dehydrogenase (EC 1.8.1.4)	2	1	1	1	1
- Citrate synthase (EC 2.3.3.1)	2	2	1	1	1
- Aconitate hydratase (EC 4.2.1.3)	1	1	1	1	1
- Isocitrate dehydrogenase [NADP] (EC 1.1.1.42)	3	2	2	2	2
- Succinyl-CoA ligase [ADP-forming] α and β chain (EC 6.2.1.5)	1	1	1	1	1
- Succinate dehydrogenase (EC 1.3.5.1)	1	1	1	1	1
- Fumarate hydratase class II (EC 4.2.1.2)	1	1	1	1	1
- Malate dehydrogenase (EC 1.1.1.37)	0	1	1	1	1
- Phosphoenolpyruvate carboxylase, archaeal (EC 4.1.1.31)	1	1	1	1	1
- NADP-dependent malic enzyme (EC 1.1.1.40)/Phosphate acetyltransferase (EC 2.3.1.8)	2	1	1	1	1
- Lactoylglutathione lyase (EC 4.4.1.5)	1	1	1	1	1
- Acetyl-coenzyme A carboxyl transferase α and β chain (EC 6.4.1.2)	2	1	1	1	1
- Fe-S protein, homolog of lactate dehydrogenase SO1521	1	1	1	1	1
**Glycerol, acetate and propionate metabolism**					
- Glycerol kinase (EC 2.7.1.30)	1	1	1	1	1
- Glycerol-3-phosphate dehydrogenase (EC 1.1.5.3)	4	4	4	4	4
- Glycerol-3-phosphate ABC transporter	1	1	1	1	1
- Glycerol dehydrogenase (EC 1.1.1.6)	1	1	1	1	1
- Glycerol-1-phosphate dehydrogenase [NAD(P)+] (EC 1.1.1.261)	3	3	4	4	2
- Acetyl-CoA synthetase (EC 6.2.1.1)	1	1	1	1	1
- Acetyl-CoA synthetase (ADP-forming) α and β chains, putative	2	1	1	1	1
- Aldehyde dehydrogenase (EC 1.2.1.3)	1	1	1	1	1
- Alcohol dehydrogenase (EC 1.1.1.1)	1	1	1	1	1
- Propionyl-CoA carboxylase beta chain (EC 6.4.1.3)	1	1	1	1	1
- Methylmalonyl-CoA epimerase (EC 5.1.99.1)	3	2	2	2	2
- Methylmalonyl-CoA mutase (EC 5.4.99.2)	1	1	1	1	1
**Respiratory chain enzymes**					
- NADH dehydrogenase (EC 1.6.99.3)	12	12	12	12	12
- NADH ubiquinone oxidoreductase chain ABCDHIJKLMN (EC 1.6.5.3)	2	2	1	1	1
Succinate dehydrogenase iron-sulfur protein (EC 1.3.5.1)	1	1	1	1	1
Succinate dehydrogenase flavoprotein subunit (EC 1.3.5.1)	1	1	1	1	1
Succinate dehydrogenase hydrophobic membrane anchor protein	1	1	1	1	1
Succinate dehydrogenase cytochrome *b* subunit	1	1	1	1	1
- Cytochrome *b*6	2	1	1	1	1
- Cytochrome P450	4	4	4	4	4
- Ferredoxin	1	1	1	1	1
- Halocyanin	3	3	3	3	3
- Cytochrome *c* oxidase polypeptide I, II and III (EC 1.9.3.1)	2	2	2	2	2
- Cytochrome *c* oxidase (B(O/a)3-type) chain I and II (EC 1.9.3.1)	1	1	1	1	1
- Cytochrome oxidase subunit I homolog	2	2	2	2	2
- Cytochrome d ubiquinol oxidase subunit I and II (EC 1.10.3.-)	10	9	9	9	9
- V-type ATP synthase subunit ABCDEFHIK (EC 3.6.3.14)	12	12	12	12	12

Several metabolic studies on halophilic archaea have shown that the Embden-Meyerhof pathway of glycolysis is incomplete due to the absence of its key enzyme, phosphofructokinase (as revealed by analysing all studied genomes so far) [1,5,16,19,20,30,39]. This key enzyme is responsible for the conversion of fructose 6-P phosphorylation to fructose 1,6-bisphosphate (F1,6BP). Halophilic archaea can use the semi-phosphorylative Entner-Doudoroff (ED) pathway for glucose degradation/assimilation. However, one can not exclude other not yet described enzymes [1]. In our studied genomes, only glucose 1-dehydrogenase and 2-dehydro-3-deoxyphosphogluconate aldolase are present. According to Bhaumik and Sonawat [60], *Halobacterium salinarum* can use a ‘reduced’ semi-phosphorylative ED pathway. In this pathway, glucose dehydrogenase catalyzes the glucose oxidation to gluconate. The studied genomes were shown deprived of gluconate dehydratase converting gluconate to 2-keto-3-deoxygluconate (KDG) and KDG kinase responsible of phosphorylation of KDG to 2-keto-3-deoxy-6-phosphogluconate (KDPG) [59,61].

All the enzyme genes necessary to the reverse EM pathway (gluconeogenesis) were found in our studied genomes. Phosphoenolpyruvate synthase which converts pyruvate to phosphoenolpyruvate, was detected. Genes coding for fructose-1,6-bisphosphatase, the key enzyme of the gluconeogenic pathway, were also found (Table 2). Fructose-1,6-bisphosphatase catalyzes the reverse reaction in gluconeogenesis by the hydrolysis of fructose 1,6-bisphosphate to fructose 6-phosphate [62]. These genes for the reverse EM pathway have also been identified in *Halobacterium* NRC-1, except for fructose-1,6-bisphosphate aldolase [39]. The complete reverse EM pathway was only found in *Halobacterium salinarum*. It is required to hexose synthesize and is essential for membrane constituents [59,63].

#### 3.3.2 Pyruvate metabolism and tricarboxylic acid cycle

All the studied strains harbored genes involved in pyruvate metabolism including 2-oxoacid ferredoxin oxidoreductase, biotin carboxylase and dihydrolipoamide dehydrogenase. The enzyme 2-oxoacid ferredoxin oxidoreductase converts generated pyruvate into acetyl-CoA which is used in the tricarboxylic acid (TCA) cycle [64]. Halophilic archaea do not encode archaeal-type pyruvate carboxylase, but they use biotin carboxylase [65]. Halophilic archaea encode dihydrolipoamide dehydrogenase which is the third component in the pyruvate dehydrogenase complex [59].

All tricarboxylic acid cycle enzymes were identified in the studied genomes, except for malate synthase and isocitrate lyase. They include citrate synthase, aconitate hydratase, isocitrate dehydrogenase, succinyl-CoA ligase, succinate dehydrogenase, fumarate hydratase and malate dehydrogenase (Table 2). Phosphoenolpyruvate carboxylase responsible for converting phosphoenolpyruvate into oxaloacetate was also detected. The phosphoenolpyruvate can be synthesized from oxaloacetate through NADP-dependent malic enzyme during gluconeogenesis. The oxaloacetate is then converted to citrate by citrate synthase.

Due to the presence of Fe-S protein, a homolog of lactate dehydrogenase (SO1521), this strain can convert pyruvate to lactate under anaerobic conditions. It is worthy to notice that lactate dehydrogenase activity has been previously demonstrated in *Halobacterium salinarum* [64,66]. The presence of lactate dehydrogenase suggests the potential for fermentative metabolism [67].

Glycerol, which is produced by eukaryotic algae *Dunaliella salina* as a compatible solute in high-salt environments, has particular significance as a nutrient for haloarchaea [66,68,69]. Additionally, dihydroxyacetone, which is produced by *Salinibacter ruber*, can also be present at high concentrations in natural salty environments and further used by haloarchaea as a nutrient [70]. The studied strains can utilize glycerol as a source of carbon and energy. Within their genomes, genes for glycerol kinase, glycerol-3-phosphate dehydrogenase, glycerol-3-phosphate ABC transport system, glycerol dehydrogenase and glycerol-1-phosphate dehydrogenase [NAD(P)+] are harboured (Table 2). Previous studies have demonstrated the role of glycerol kinase in phosphorylating glycerol to glycerol-3-phosphate (G3P) [16]. Glycerol-3-phosphate dehydrogenase is used for the oxidation of G3P to dihydroxyacetone phosphate (DHAP) [71]. Glycerol can also be converted to DHAP by glycerol dehydrogenase [30]. Glycerol-1-phosphate dehydrogenase catalyzes the reversible conversion between DHAP and Glycerol-1P [72].

Acetate, known as a major carbon source in hypersaline environments, plays a role in the haloarchaea nutrition [73]. An ADP-forming acetyl-CoA synthetase which is responsible of acetate formation from acetyl-CoA was detected. This enzyme is involved in acetate formation and ATP synthesis [25]. The presence of genes coding for acetyl-CoA synthetase in our sequenced genomes indicates the potential conversion of acetate into acetyl-CoA, which can enter the methylaspartate cycle for gluconeogenesis or the TCA cycle for energy production [16,26]. Acetate can be converted, by aldehyde dehydrogenase, to acetaldehyde which is further converted to ethanol by alcohol dehydrogenase.

Propionate can be used as the sole carbon source by the studied strains, as they have the genes encoding the necessary enzymes for converting propionate to succinyl-CoA. These enzymes include acetyl-CoA synthetase, propionyl- CoA carboxylase beta chain, methylmalonyl-CoA epimerase, and methylmalonyl-CoA mutase as demonstrated previously [16,30].

**Fig. 3:**
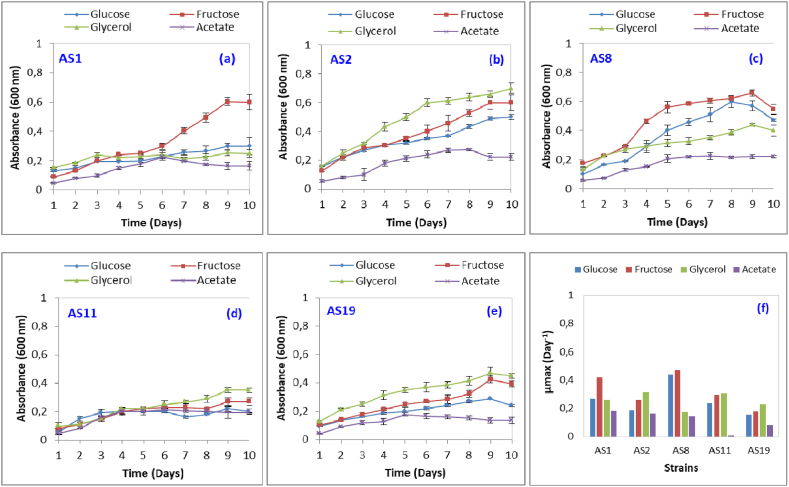
Growth kinetics (a, b, c, d and e) and growth rate (f) of the five archaeal strains (AS1, AS2, AS8, AS11 and AS19) with different carbon source (glucose, fructose, glycerol and acetate). Values are means of three replications ± SD.

In summary, these results revealed the genomic potential of the five strains to utilize glucose, fructose, acetate and glycerol as a source of carbon and energy. To evaluate their capacity to use the carbon source for growth, they were cultured for 10 days in minimal media supplemented by glucose, fructose, glycerol or acetate at a concentration of 10 g/L (Fig. 3). Optical density-based assay results suggest that acetate was the lowest-used carbon source. Fructose was the best carbon source for the strains AS1 (μmax of 0.418 day^−1^) and AS8 (μmax of 0.472 day^−1^), followed by glucose and glycerol. For the strains AS2, AS11 and AS19, glycerol was the preferred carbon source followed by fructose and glucose. Contrary to that found in the present work, *Halobacterium salinarum* NRC-1 was demonstrated enable to metabolize glucose, despite the presence of a complete semi-phosphorylative Entner-Doudoroff pathway [23,39].

### 3.4 Respiratory chain

Our genomes appeared to contain all the genes required for a cytochrome c-containing respiratory chain. Oxidative phosphorylation occurs through five mitochondrial complexes: complex I (NADH dehydrogenase), complex II (succinate dehydrogenase), complex III (cytochrome *b*6, P450, ferredoxin and halocyanin), complex IV (cytochrome oxidase) and complex V (V-type ATP synthase) (Table 2). Complex I, represented by NADH dehydrogenases and the NADH ubiquinone oxidoreductase chain (ABCDHIJKLMN), is responsible for NADH oxidation [26]. According to Favreau et al. [38], the NADH oxidoreductase chain of *Halobacterium salinarum* NRC-1, which can survive in brine inclusion within salt crystals, was composed of only 4 subunits B, C, D and I. In addition to NADH dehydrogenases, the studied archaeal strains are predicted to encode numerous genes for complex II, represented by succinate dehydrogenase (subunit, cytochrome *b*, iron-sulfur protein and flavoprotein). Complex III, represented by cytochrome *b*6, P450, ferredoxin and halocyanin, is required to transfer electrons [27]. Genes coding for halocyanins and ferredoxin were also detected in the genomes. Halocyanin acts as an electron carrier to the terminal oxidases in halophilic archaea and is mainly associated to anaerobic processes like denitrification [[73], [74], [75], [76]]. In the analyzed genomes, complex IV was represented by many genes coding for cytochrome *c* oxidase polypeptide I, II and III, cytochrome oxidase subunit I homolog, cytochrome d, ubiquinol oxidase subunits I and II and cytochrome *c* oxidase (B(O/a)3-type) chain I and II. Cytochrome *c* oxidases, allowing respiration under low-O_2_ levels, accept electrons from a variety of donors and reduce dioxygen to water [26,77]. Complex V was represented by V-type ATP synthase ABCDEFHIK. According to Talaue et al. [78], V-type ATP synthase produces ATP from ADP. Non-of the genes related to denitrification (as potential anaerobic respiratoy pathway) have been identified.

### 3.5 Anaerobic metabolism

The five studied annotated genomes conducted by RAST revealed the presence of genes involved in anaerobic metabolism. Specifically, genes coding for a dimethyl sulfoxide reductase and trimethylamine N-oxide reductase were identified (Table S3). Previous research has reported the utilization of DMSO for anaerobic respiration in different haloarchaeal strains across differents genera of haloarchaeal strains such as *Haloarcula*, *Haloferax* and *Halobacterium* [34,79]. More recently, the anaerobic respiration ability has also been observed in members of *Halodesulfurarchaeum* genus [80]. The studied archaeal strains also demonstrated their ability to ferment arginine by arginine deiminase pathway, like other *Halobacterium* genera. Recent proteomic analysis of *Halobacterium salinarum* NRC-1 highlighted the role of enzymes involved in arginine deiminase pathway, which includes arginine deiminase, ornithine carbamoyltransferase, carbamate kinase. Additionally, this analysis revealed the presence of two other enzymes argininosuccinate lyase and argininosuccinate synthase, further enhancing our understanding of this metabolic pathway [37].

As recently demonstrated, the studied strains have genes for the biosynthesis of the bacteriorhodopsin protein and retinal [4]. They are phototrophs and can use light energy absorbed by retinal-based pigments, as many other halophilic archaea, such as *Haloquadratum* [81] and *Halobacterium* [40]. Like other *Halobacterium* species, the strains can survive in extreme environments by changing their energetic metabolism with a preference for aerobic respiration. They were also able to survive in phototrophy, arginine fermentation and anaerobic respiration using dimethyl sulfoxide (DMSO) and trimethylamine oxide (TMAO) [37,64,82,83]. Three genes coding for thiosulfate sulfurtransferase, sulfite oxidase homolog and conserved hypothetical protein probably involved in respiration and assimilation of sulfate were identified in the genomes. These findings suggest that halophilic archaea possess genes coding for the intermediate reduction of sulfur-containing molecules [34,84].

### 3.6 Pentose phosphate pathway and ribose production

All the strains have a likely 6-phosphogluconate dehydrogenase, (Table S4), the pivotal enzyme within the oxidative pentose phosphate pathway, responsible for converting gluconate 6-phosphate into ribulose 5-phosphate, a crucial nucleic acid precursor, through oxidative decarboxylation [85]. Enzymes responsible for the initial dehydrogenation of glucose 6-P to glucono-1,5-lactone 6-phosphate, as well as its subsequent hydrolysis to gluconate 6-phosphate were not identified. According to Gonzalez et al. [45], genes for the first two steps in *Halobacterium salinarum* R-1 could not be assigned, but experimental evidence supports the presence of the enzyme “glucono-1,5-lactone 6-phosphate”. According to Falb et al. [64], an operative modified oxidative pentose phosphate pathway is suggested for halophilic archaea.

Genomic analysis of the strains revealed the presence of a gene encoding a ribose-1,5-bisphosphate isomerase homolog that was annotated as “Translation initiation factor 2B”. The protein coded by such gene plays a crucial role in converting ribulose 5-phosphate into ribose 5-phosphate [28]. Furthermore, the presence of ribose-phosphate pyrophosphokinase, which is responsible of the ribose-5-phosphate conversion to phosphoribosyl pyrophosphate (PRPP) was identified. This conversion is a pivotal step in purine and pyrimidine nucleotides biosynthesis. This reaction links the pentose phosphate pathway to the nucleotide biosynthesis [86]. In addition to these findings, we retrieved in the studied genomes, enzymes such as deoxyribose-phosphate aldolase and ribokinase. These enzymes facilitate the formation of 2-Deoxy-D-ribose-5P from glyceraldehyde 3-phosphate and 2-Deoxy-D-ribose respectively. A adenine phosphoribosyltransferase, which is responsible for converting phosphoribosyl pyrophosphate (PRPP) and adenine into AMP [64], was also identified. Numerous genes involved in purines and pyrimidines synthesis that is crucial for DNA and RNA synthesis were revealed in the studied genomes (Table S4). IMP cyclohydrolase domain is responsible for converting 5-aminoimidazole-4-carboxamide ribonucleotide to IMP [87]. Genes annotated as “uridine phosphorylase” have a function of guanosine phosphorylase as demonstrated by Sato et al. [28]. This diverse array of enzymes and genes underscores the comprehensive capabilities of these archaeal genomes in nucleotide biosynthesis and metabolism.

### 3.7 Amino acids biosynthesis

The genome sequences analysis of the studied archaeal strains showed the presence of several genes coding for many amino acid biosynthesis enzymes (Table S5). Glutamate and glutamine can be synthesized from the TCA cycle intermediate “2-oxoglutarate” by aspartate aminotransferase. Glutamate dehydrogenase (GDH) and glutamine synthetase/glutamate synthase cycle (GS/GOGAT cycle) are the key enzymes for the synthesis of these two amino acids as well as for the ammonium assimilation in several microorganisms including *Halobacterium salinarum* [74,88,89]. Aspartate can be derived from oxaloacetate (TCA cycle intermediate) by aspartate aminotransferase. According to Bhaumik and Sonawat [66], aspartate aminotransferase can be involved in alanine synthesis through pyruvate transamination in *Halobacterium*. Aspartate can be converted to asparagine-by-asparagine synthetase and oxidized to iminoaspartate by L-aspartate oxidase [64]. The archaeal strains have an ornithine cyclodeaminase and proline dehydrogenase. The ornithine cyclodeaminase allows ornithine to be broken down into proline and NH_3_. The proline dehydrogenase has a role in proline oxidation and cellular redox control [16].

All five archaeal strains possess genes for phosphoglycerate dehydrogenase responsible for the conversion of glycerate 3-phosphate, a glycolytic intermediate to phosphoserine and serine [64]. Serine enters central metabolism as pyruvate, through the action of threonine dehydratase [16]. Threonine can be converted by threonine dehydratase to 2-oxobutanoate and then to propanoyl-CoA. A threonine dehydratase required to synthesize the isoleucine precursor 2-oxobutyrate, is encoded in the 5 archaeal strains. Glycine can be potentially derived from serine by serine hydroxymethyltransferase and from threonine by threonine aldolase. A gene encoding components of the glycine cleavage system P2 protein (glycine dehydrogenase) was found in the 5 genomes. The gene coding for “5-methyltetrahydropteroyltriglutamate-homocysteine methyltransferase”, responsible of homocysteine conversion to methionine, was also found. Histidine was predicted to be synthetized by 2 genes coding for histidinol-phosphatase and imidazoleglycerol-phosphate dehydratase (EC 4.2.1.19) [74]. The archaeal strains harbored the genes required for tryptophan synthesis like tryptophan synthase α and β chain. Phenylalanine, tyrosine and tryptophan can be synthesized by the five strains. Many enzymes involved in shikimate pathway for 3-dehydroquinate synthesis like 2-amino-3,7-dideoxy-D-threo-hept-6-ulosonate synthase, 3-dehydroquinate synthase type II and 3-dehydroquinate dehydratase I [90] were found (Table S5).

The synthesis pathways of many amino acids were predicted through strains genome annotation, including glutamate, glutamine, aspartate, alanine, asparagine, proline, serine, histidine, tryptophan, phenylalanine, threonine, glycine, methionine and tyrosine. The enzymes for biosynthesis of valine, leucine, isoleucine, lysine and arginine were absent. This result has been proven elsewhere [74]. Previous studies showed that *Halobacterium* NRC-1 has no biosynthetic capabilities for threonine, glycine, methionine and tyrosine [64]. Glutamate can be degraded in all the studied genomes to mesaconate by methylaspartate mutase and methylaspartate ammonia-lyase. Genes coding for pyruvoyl-dependent arginine decarboxylase and agmatinase were found in the genomes. These two genes have been recently evidenced in *Halobacterium* genomes [6]. They are responsible for the use of arginine for the biosynthesis of polyamines for nucleosome maintenance [91]. Two genes coding for kynureninase and L-tyrosine decarboxylase having a role in aromatic amino acid degradation [92] were found in the genomes.

### 3.8 Genes of biotechnological interest

The analysis of the sequences revealed a pool of genes with potential applications in biotechnology (mainly plant biotechnology, synthesis of vitamins highly marketed and synthesis of natural pigments like carotenoids which are highly demanded by industries like pharmacy, agrifood and cosmetics). The following sections display in details the potential applications of those genes.

#### 3.8.1 Plant growth promotion genes

The genomic analysis confirmed the presence of genes associated with plant growth properties as detailed in Table 3. Genes encoding for phosphate solubilization enzymes, like inorganic pyrophosphatase and alkaline phosphatase, were observed in the studied genomes. These enzymes facilitate the hydrolysis of inorganic phosphate converting it into an accessible form of phosphorus to plants [93]. In addition, the analysis revealed the presence of genes involved in the uptake and transport of phosphate, known as PstSCAB and phoU [94]. Additionally, the strain genomes were found to harbour numerous genes involved in iron metabolism and siderophore production. These genes include several ferric iron ABC transporters, iron-dependent repressors and a cluster of six genes encoding siderophore biosynthesis, such as monooxygenase, diaminobutyrate decarboxylase, diaminobutyrate--2-oxoglutarate aminotransferase and two domains acting as acyltransferase and siderophore synthetase. Siderophores can chelate iron from environments [95,96]. Genomic analysis revealed also the presence of genes associated with the tryptophan synthetic pathway, which code for enzymes involved in the biosynthesis of the indole acetic acid (IAA) including tryptophan synthase (alpha and beta chains), indole-3-glycerol phosphate synthase, tryptophanyl-tRNA synthetase, anthranilate synthase (comprising aminase and amido transferase components), phosphoribosyl anthranilate isomerase and anthranilate phosphoribosyltransferase. Such genes were recently identified in halophilic bacteria by Sharma et al. [94]. Indole acetic acid (IAA) has a crucial role in plant growth and development [97]. A gene associated with phenazine production was also predicted. This later is a natural bacterial antibiotic able to protect plants from disease [98]. Genes involved in ammonium assimilation, including glutamine synthetase type1, citrate synthase, glutamate dehydrogenase, CTP synthase, and asparagine synthetase, as recently described by Sharma et al. [94], were retrieved. Numerous genes related to mobility were also detected including those associated with flagella biosynthesis (FlaEFGHJ, Flp pilus assembly protein TadC, prepilin\preflagellin peptidase type IV and Flagellin FlaB2) and chemotaxis (CheY, CheA, CheB, CheC, CheD and CheR). According to Palma et al. [99], the strains motility allows them to move, colonize, and spread in plants.

**Table 3:** Genes related to plant growth promotion.

Genes names	Strain				
	AS1	AS2	AS8	AS11	AS19
**Phosphate solubilization**					
- Inorganic pyrophosphatase (EC 3.6.1.1)	1	1	1	1	1
- Alkaline phosphatase (EC 3.1.3.1)	1	1	1	1	1
- Phosphate transport system regulatory protein PhoU	1	1	1	1	1
- Probable low-affinity inorganic phosphate transporter	1	2	1	1	1
- Phosphate ABC transporter, substrate-binding protein PstS (TC 3.A.1.7.1)	1	1	1	1	1
- Phosphate ABC transporter, permease protein PstC (TC 3.A.1.7.1)	1	1	1	1	1
- Phosphate ABC transporter, permease protein PstA (TC 3.A.1.7.1)	2	2	2	2	2
- Phosphate ABC transporter, ATP-binding protein PstB (TC 3.A.1.7.1)	2	2	3	2	2
- Phosphate regulatory protein homolog	2	2	2	2	2
**Siderophore production**					
- Ferric iron ABC transporter, permease protein	2	1	1	1	1
- Ferric iron ABC transporter, iron-binding protein	1	1	1	1	1
- ABC transporter, substrate-binding protein (cluster 1, maltose/g3p/polyamine/iron)	2	2	3	2	2
- ABC transporter, ATP-binding protein (cluster 1, maltose/g3p/polyamine/iron); ABC transporter, ATP-binding protein (cluster 10, nitrate/sulfonate/bicarbonate)	1	1	1	1	1
- ABC transporter, substrate-binding protein (cluster 8, B12/iron complex)	2	2	2	2	2
- ABC transporter, ATP-binding protein (cluster 8, B12/iron complex)	2	1	1	1	1
- Iron-dependent repressor	3	3	3	3	3
- Siderophore biosynthesis protein, monooxygenase	1	1	1	1	1
- Siderophore biosynthesis L-2,4-diaminobutyrate decarboxylase	1	1	1	1	1
- Siderophore biosynthesis diaminobutyrate--2-oxoglutarate aminotransferase (EC 2.6.1.76)	1	1	2	1	1
- Siderophore synthetase large component, acetyltransferase	1	2	1	1	1
- Siderophore synthetase small component, acetyltransferase	1	1	1	1	1
- Siderophore synthetase component, ligase	1	1	1	1	1
**Indole acetic acid (IAA) biosynthesis**					
- Tryptophan synthase alpha chain (EC 4.2.1.20)	1	1	1	1	1
- Tryptophan synthase beta chain (EC 4.2.1.20)	1	1	1	1	1
- Indole-3-glycerol phosphate synthase (EC 4.1.1.48)	1	1	1	1	1
- Tryptophanyl-tRNA synthetase	4	3	2	2	2
- Phosphoribosyl anthranilate isomerase (EC 5.3.1.24)	1	1	1	1	1
- Phosphoribosyl formimino-5-aminoimidazole carboxamide ribotide isomerase (EC 5.3.1.16)	2	1	1	1	1
- Anthranilate phosphoribosyltransferase (EC 2.4.2.18)	1	1	1	1	1
- Anthranilate phosphoribosyltransferase-like protein	1	1	1	1	1
- Anthranilate synthase, aminase component (EC 4.1.3.27)	1	1	1	1	1
- Anthranilate synthase, amidotransferase component (EC 4.1.3.27)	1	1	1	1	1
- Aminodeoxychorismate lyase (EC 4.1.3.38)	1	1	1	1	1
- Para-aminobenzoate synthase, aminase component (EC 2.6.1.85)	1	1	1	1	1
- Para-aminobenzoate synthase, amidotransferase component (EC 2.6.1.85)	1	1	1	1	1
**Phenazine biosynthesis protein PhzF like**	1	1	1	1	1
**Ammonium assimilation**					
- Glutamine synthetase type I (EC 6.3.1.2)	1	3	1	1	1
- Citrate synthase	2	1	1	1	1
- Glutamate dehydrogenase	2	3	3	3	3
- CTP synthase (EC 6.3.4.2)	2	1	1	1	1
- Asparagine synthetase	1	1	1	1	1
**Cell motility**					
- Flagella-related protein FlaEFGHJ	5	5	5	5	5
- Flagella-related ATPase FlaI	1	1	1	1	1
- Flp pilus assembly protein TadC	1	1	1	1	1
- Flagellin FlaB2	2	1	1	2	3
- Signal peptidase, type IV - prepilin/preflagellin	1	1	1	1	1
**Chemotaxis**					
- Chemotaxis regulator-transmits chemoreceptor signals to flagellar motor components CheY	1	1	1	1	1
- Positive regulator of CheA protein activity (CheW)	2	2	2	2	2
- Signal transduction histidine kinase CheA	1	1	1	1	1
- Chemotaxis response regulator protein-glutamate methylesterase CheB (EC 3.1.1.61)	1	1	1	1	1
- Chemotaxis protein CheC -- inhibitor of MCP methylation	2	2	2	2	2
- Chemotaxis protein CheD	1	1	1	1	1
- Chemotaxis protein methyltransferase CheR (EC 2.1.1.80)	1	1	1	1	1
- Methyl-accepting chemotaxis protein	1	1	1	1	1

#### 3.8.2 Vitamins

The genomic analysis of the five archaeal strains revealed their genetic capacity for cobalamin (vitamin B_12_) biosynthesis. Cobalamin, an essential vitamin widely used in medical and food industries, is exclusively produced by many bacteria and archaea [100]. It can be synthesized through aerobic or anaerobic pathways, starting from uroporphyrinogen III as the precursor [101]. Genes only involved in anaerobic pathway for cobalamin biosynthesis were identified (Table 4). This specific pathway was recently identified through the genomic investigation in *Halobacterium salinarum* [6], and in other haloarchaeal members of the genera *Halonotius* [1] and *Halomicroarcula* [5]. The missing genes from the anaerobic pathway were not detected in any of the studies aiming at the characterization of archaeal cobalamin producers [1,27]. According to Doxey et al. [102], these genes are not essential for this pathway.

**Table 4:** Biotechnologically potential genes.

Genes names	Strain				
	AS1	AS2	AS8	AS11	AS19
**Cobalamin (Vitamin B**_**12**_**) biosynthesis**					
- Glutamyl-tRNA synthetase (EC 6.1.1.17)	2	1	1	1	1
- Glutamyl-tRNA reductase (EC 1.2.1.70)	1	1	1	1	1
- Glutamate-1-semialdehyde 2,1-aminomutase (EC 5.4.3.8)	1	1	1	1	1
- Porphobilinogen synthase (EC 4.2.1.24)	1	1	1	1	1
- Porphobilinogen deaminase (EC 2.5.1.61)	1	1	1	1	1
- Uroporphyrinogen-III synthase (EC 4.2.1.75)	1	1	1	1	1
- Uroporphyrinogen-III methyltransferase (EC 2.1.1.107)	1	1	1	1	1
- Precorrin-2 oxidase (EC 1.3.1.76)	1	1	1	1	1
- Sirohydrochlorin cobaltochelatase CbiX(long) (EC 4.99.1.3)	1	1	1	1	1
- Cobalt-precorrin-2 C(20)-methyltransferase (EC 2.1.1.151)	1	1	1	1	1
- Cobalt-precorrin-3 C(17)-methyltransferase (EC 2.1.1.272)	2	2	2	2	2
- Cobalt-precorrin 5A hydrolase (EC 3.7.1.12)	1	1	1	1	1
- Cobalt-precorrin-6B C15-methyltransferase [decarboxylating] (EC 2.1.1.196)	1	1	1	1	1
- Cobalt-precorrin-7 (C5)-methyltransferase (EC 2.1.1.289)	1	1	1	1	1
- Cobalt-precorrin-8 methylmutase (EC 5.4.99.60)	1	1	1	1	1
- Cobyrinic acid a,c-diamide synthetase (EC 6.3.5.11)	1	1	1	1	1
- Cob(I)alamin adenosyltransferase (EC 2.5.1.17)	1	2	2	2	2
- Cobyric acid synthase (EC 6.3.5.10)	2	1	1	1	1
- Adenosylcobinamide-phosphate synthase (EC 6.3.1.10)	2	1	1	1	1
- Predicted adenosylcobinamide-phosphate guanylyltransferase CobY (EC 2.7.7.62)	2	1	1	1	1
- Cobalamin synthase (EC 2.7.8.26)	1	1	1	1	1
- L-threonine 3-O-phosphate decarboxylase (EC 4.1.1.81)	1	2	1	1	1
- Nicotinate-nucleotide-dimethylbenzimidazole phosphoribosyltransferase (EC 2.4.2.21)	1	1	1	1	1
**Folate (Vitamin B**_**9**_**) biosynthesis**					
- 6-carboxy-5,6,7,8- tetrahydropterin synthase (EC 4.1.2.50)	1	1	1	1	1
- 7-carboxy-7-deazaguanine synthase (EC 4.3.99.3)	1	1	1	1	1
- 7-cyano-7-deazaguanine synthase (EC 6.3.4.20)	1	1	1	1	1
- GTP cyclohydrolase MptA (EC 3.5.4.39)	1	1	1	1	1
- Molybdopterin adenylyltransferase (EC 2.7.7.75)	1	1	1	1	1
- Molybdenum cofactor guanylyltransferase (EC 2.7.7.77)	1	1	1	1	1
- Dihydrofolate synthase (EC 6.3.2.12) @ Folylpolyglutamate synthase (EC 6.3.2.17)/Alternative dihydrofolate reductase 2/Dihydropteroate synthase (EC 2.5.1.15)	2	1	1	1	1
- Aminodeoxychorismate lyase (EC 4.1.3.38)	1	1	1	1	1
**Biotin (Vitamin B**_**7**_**) biosynthesis**					
- Biotin operon repressor/Biotin--protein ligase (EC 6.3.4.9)(EC 6.3.4.10)(EC 6.3.4.11)(EC 6.3.4.15)	1	1	1	1	1
- Transmembrane component BioN of energizing module of biotin ECF transporter	2	1	2	1	1
- ATPase component BioM of energizing module of biotin ECF transporter	1	1	1	1	1
- Substrate-specific component BioY of biotin ECF transporter	1	1	1	1	1
- Acetyl-coenzyme A carboxyl transferase alpha chain (EC 6.4.1.2)/Acetyl-coenzyme A carboxyl transferase beta chain (EC 6.4.1.2)	1	1	1	1	1
**Pantothenate (Vitamin B**_**5**_**) biosynthesis**					
- Pantothenate:Na + symporter (TC 2.A.21.1.1)	1	1	1	1	1
- Phosphopantothenate synthetase, archaeal (EC 6.3.2.36)	1	1	1	1	1
- 2-dehydropantoate 2-reductase (EC 1.1.1.169)	1	1	1	1	1
- Pantoate kinase, archaeal (EC 2.7.1.169)	1	1	1	1	1
**Nicotinate and nicotinamide (Vitamin B**_**3**_**) biosynthesis**					
- Nicotinate phosphoribosyltransferase (EC 6.3.4.21)	2	1	1	1	1
- Nicotinate-nucleotide--dimethylbenzimidazole phosphoribosyltransferase (EC 2.4.2.21)	1	1	1	1	1
− 5'-nucleotidase SurE (EC 3.1.3.5)	1	1	1	1	1
- Nicotinamide-nucleotide amidase (EC 3.5.1.42)	1	1	1	1	1
- NAD synthetase (EC 6.3.1.5)	1	1	1	1	1
- NAD kinase (EC 2.7.1.23)	1	1	1	1	1
- Succinate-semialdehyde dehydrogenase [NAD] (EC 1.2.1.24); Succinate-semialdehyde dehydrogenase [NADP+] (EC 1.2.1.79)	2	2	1	1	1
- Nicotinamide-nucleotide adenylyltransferase, NadM family (EC 2.7.7.1)	1	1	1	1	1
- Quinolinate phosphoribosyltransferase [decarboxylating] (EC 2.4.2.19)	1	1	1	1	1
- Quinolinate synthetase (EC 2.5.1.72)	1	1	1	1	1
**Riboflavin (Vitamin B**_**2**_**) biosynthesis**					
- Riboflavin synthase eubacterial/eukaryotic (EC 2.5.1.9)	1	0	0	0	0
− 7,8-didemethyl-8-hydroxy-5-deazariboflavin synthase subunit 1 and 2	2	2	2	2	2
- DNA-binding HTH domain in riboflavin kinase/CTP-dependent archaeal riboflavin kinase (EC 2.7.1.161)	1	1	1	1	1
− 2,5-diamino-6-ribosylamino-pyrimidinone 5-phosphate reductase, fungal/archaeal (EC 1.1.1.302)	2	1	1	1	1
- GTP cyclohydrolase III (EC 3.5.4.29)	2	2	2	2	2
- FMN adenylyltransferase, type 3 archaeal (EC 2.7.7.2)	1	1	1	1	1
**Thiamine (Vitamin B**_**1**_**) biosynthesis**					
- Thiamine-monophosphate kinase (EC 2.7.4.16)	1	1	1	1	1
- Hydroxymethylpyrimidine phosphate kinase ThiD (EC 2.7.4.7)/Thiamin-phosphate synthase ThiN (EC 2.5.1.3)	2	1	1	1	1
- tRNA 4-thiouridine synthase (EC 2.8.1.4)	1	1	2	1	1
**Menaquinone (Vitamin K**_**2**_**) biosynthesis**					
- Isochorismate synthase (EC 5.4.4.2)	1	1	1	1	1
- 2-succinyl-5-enolpyruvyl-6-hydroxy-3-cyclohexene-1-carboxylic-acid synthase (EC 2.2.1.9)	1	1	1	1	1
- O-succinylbenzoate synthase (EC 4.2.1.113)	2	2	1	1	1
- O-succinylbenzoic acid--CoA ligase (EC 6.2.1.26)	1	1	1	1	1
- Naphthoate synthase (EC 4.1.3.36)	0	1	1	1	1
− 1,4-dihydroxy-2-naphthoate polyprenyltransferase (EC 2.5.1.74)	1	1	1	1	1
- Demethylmenaquinone methyltransferase (EC 2.1.1.163)	1	1	0	1	1
- NADH-ubiquinone oxidoreductase chain N (EC 1.6.5.3)	1	1	1	1	1
- Ubiquinone/menaquinone biosynthesis methyltransferase UbiE/COQ5 (EC 2.1.1.-)	1	1	1	1	1

The genomic analysis highlighted the ability of the studied strains to produce folate (vitamin B_9_). Folate plays an essential role in cell metabolism, including DNA/RNA biosynthesis, synthesis of nucleotides and some amino acids. It is renowned for its antioxidant properties which protect the genome [103]. Halophilic archaea, capable of producing folate, hold promise for enhancing natural folate content in salted food products, as indicated by Fontana et al. [6]. The archaeal genomes exhibited the presence of biosynthesis pathways for various other essential vitamins, including biotin (vitamin B_7_), pantothenate (vitamin B_5_), nicotinate and nicotinamide (vitamin B_3_), riboflavin (vitamin B_2_) and thiamine (vitamin B_1_) as outlined in Table 3. These vitamins serve as coenzymes in numerous biochemical reactions and are involved in various metabolic pathways [104,105]. Additionally, genes related to menaquinone biosynthesis, also known as vitamin K_2_, were discovered in the genomes. In *Halobacterium salinarum*, menaquinone has been proposed to function as an electron carrier, shuttling electrons from complex I to a complex III analogue in the respiratory chain [73,74]. According to Kellermann et al. [106], menaquinones were the most constituent of the *Halobacterium salinarum* membrane. The capacity of the strains to synthesize vitamins implies their global biogeochemical, metabolic and ecological importance as highlighted by Karner et al. [107] and Durán-Viseras et al. [1].

#### 3.8.3 Carotenoids

The genomic analysis of the five archaeal strains revealed their capacity for carotenogenesis as expected due to the colour of the cells (pale pink to red). Among the genes identified those involved in the mevalonate pathway, isoprene and carotenoid biosynthesis are included (Table S6).

##### 3.8.3.1 Mevalonate pathway

Upon analysis of the archaeal strain's genomes, six genes coding for enzymes of mevalonate pathway were found including acetyl-CoA acetyltransferase (EC 2.3.1.9), hydroxymethylglutaryl-CoA synthase (EC 2.3.3.10), hydroxymethylglutaryl-CoA reductase (EC 1.1.1.34), mevalonate kinase (EC 2.7.1.36), phosphomevalonate decarboxylase (EC 4.1.1.99) and isopentenyl phosphate kinase (EC 2.7.4.26). According to Moise et al. [108] and Zuo et al. [109], acetyl-CoA acetyltransferase is responsible for the condensation of two acetyl-CoA molecules to form an acetoacetyl-CoA. Then, an acetyl-CoA is added to acetoacetyl-CoA to generate 3-hydroxy-3-methylglutaryl- CoA (HMG-CoA) by hydroxymethylglutaryl-CoA synthase. HMG-CoA is converted to mevalonate by hydroxymethylglutaryl-CoA reductase. Then, mevalonate is phosphorylated to phosphomevalonate by mevalonate kinase, decarboxylated by phosphomevalonate decarboxylase and then pyrophosphorylated by isopentenyl phosphate kinase, to produce isopentenyl-diphosphate (IPP) [64,110].

As demonstrated previously by Giani et al. [110], through the analysis of more than 100 genomes of many halophilic archaea genera (*Haloarcula*, *Haloferax*, *Halorubrum*, *Haloterrigena*, *Haloquadratum*, *Natronobacterium* and *Natronomonas*), the studied archaeal strains use mevalonate pathway. Giani et al. [110] confirmed the presence and the role of two enzymes of the mevalonate pathway that are often absent in archaeal genomes: isopentenyl phosphate kinase and phosphomevalonate decarboxylase. These two enzymes carry out the two activities necessary to provide isopentenyl diphosphate (IPP) in haloarchaea [29,111].

##### 3.8.3.2 Isoprene biosynthesis

The genome analysis revealed that the strains have 3 genes coding for enzymes of isoprene biosynthesis (Table S6), except two enzymes: farnesyl diphosphate synthase (2.5.1.10), responsible for IPP conversion to farnesyl pyrophosphate (FPP) [112] and (2.5.1.1) involved in IPP conversion to geranyl-diphosphate (GPP) [110]. Carotenoids are formed from isopentenyl diphosphate (IPP, C5) and its isomer dimethylallyl diphosphate (DMAPP, C5), both derived from mevalonate pathway like other previous studies [108,109]. Isopentenyl-diphosphate delta-isomerase (EC 5.3.3.2) is responsible for IPP conversion to DMAPP.

Geranylgeranyl diphosphate synthase (2.5.1.29) (CrtE) is involved in the conversion of DMAPP to GPP after the addition of IPP sequences. It is also implicated in the addition of IPP molecule in the GPP to form farnesyl-diphosphate (FPP) (15 carbons) [112,113]. A geranylgeranyl diphosphate synthase (2.5.1.31) was also detected. It is implicated in the addition of IPP molecule to form geranylgeranyl-diphosphate (GGPP) (20 carbons) [110].

##### 3.8.3.3 Carotenogenic pathway

The genome analysis revealed the presence of genes encoding key enzymes required in carotenoid biosynthesis including phytoene synthase (*CrtB*), phytoene desaturase (*CrtI*), phytoene dehydrogenase, lycopene elongase (*LyeJ*) and lycopene cyclase (*CrtY*).

The first step of carotenoid biosynthesis is the formation of phytoene after condensation of two molecules of geranylgeranyl diphosphate (GGPP) (C20 molecule) by an enzyme called phytoene synthase encoded by the *CrtB* (EC 2.5.1.32) [29,110,114]. Lycopene is generated from phytoene, through a series of desaturations and isomerizations that increase the number of conjugated double-bonds, catalyzed by phytoene desaturase encoded by *CrtI* (EC 1.14.99) [112,114,115]. Genes encoding phytoene dehydrogenase (EC 1.14.99) is also responsible for lycopene synthesis [53]. Lycopene elongase (*LyeJ*) is known to catalyze the committed step in bacterioruberin biosynthesis [114,116]. It converts lycopene into dihydroisopentenyldehydrorhodopin (DH-IDR) [112]. Phytoene desaturase (*CrtI*) is involved in various steps of the bacterioruberin biosynthesis pathway as described previously by Mishra et al. [113]. It converts DH-IDR into isopentenyldehydrorhodopin (IDR), which is converted to dihydrobisanhydrobacterioruberin (DH-BABR) by lycopene elongase (*LyeJ*). DH-BABR is converted to bisanhydrobacterioruberin (BABR) by *CrtI* [114]. A previous study regarding the carotenoid biosynthesis in *Halobacterium salinarum* revealed the role of *LyeJ* gene in the DH-IDR conversion to tetrahydrobisanhydrobacterioruberin (TH-BABR) [108]. The gene (*CruF*), responsible for the final step in bacterioruberin biosynthesis in various halophilic archaea [114], was not found in the studied strains. Comparative genomic analysis of halophilic archaea from coastal biosystems detected *crtI* in 64.66 % of genomes, while *cruF* was observed in only 40.75 % of archaea [53,117].

**Fig. 4:**
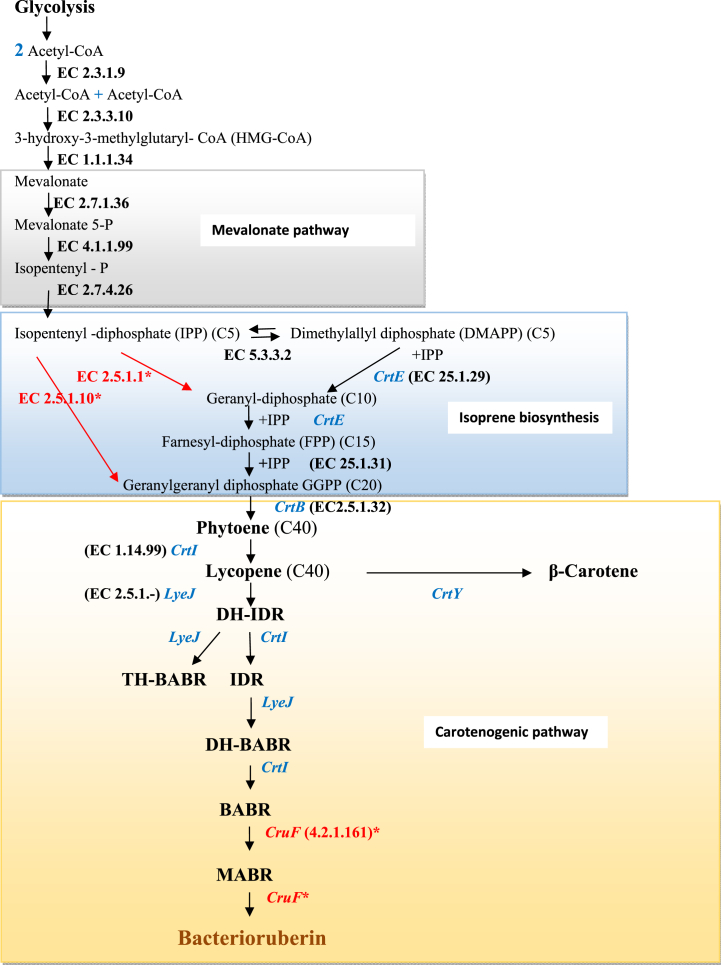
Proposed main steps in the mevalonate, isoprene and carotenoid biosynthesis pathways of the five archaeal strains. Acetyl-CoA: Acetyl-Coenzyme A; Mevalonate 5-P: Mevalonate 5-Phosphate; Isopentenyl-P: Isopentenyl-Phosphate; DH-IDR: Dihydroisopentenyldehydrorhodopsin; IDR: Isopentenyldehydrorhodopsin; DH-BABR: Dihydrobisanhyrobacterioruberin; BABR: Bisanhydrobacterioruberin; TH-BABR: Tetrahydrobisanhyro-bacterioruberin; MABR: Monoanhydrobacterioruberin; **EC 2.3.1.9**: Acetyl-CoA acetyltransferase; **EC 2.3.3.10**: Hydroxymethylglutaryl-CoA synthase; **EC 1.1.1.34**: Hydroxymethylglutaryl-CoA reductase; **EC 2.7.1.36**: Mevalonate kinase; **EC 4.1.1.99**: Phosphomevalonate decarboxylase; **EC 2.7.4.26**: Isopentenyl phosphate kinase; **EC 5.3.3.2**: Isopentenyl-diphosphate delta-isomerase; **EC 25.1.29 (*Crt E*)**: Geranylgeranyl diphosphate synthase**EC 2.5.1.31**: Undecaprenyl diphosphate synthase; **EC2.5.1.32** (***CrtB***): Phytoene synthase; **EC 1.14.99** (***CrtI***): Phytoene desaturase; **EC 2.5.1** (***LyeJ***): Lycopene elongase and ***CrtY***: Lycopene cyclase. **EC 2.5.1.1∗**: dimethylallyltransferase; **EC 2.5.1.10∗**: geranyltranstransferase and **4.2.1.161 (*CruF*)∗**: Carotenoid hydratase are absent (Red color).

All the analyzed genomes have at least one copy of genes coding for the following enzymes: phytoene synthase, phytoene desaturase, phytoene dehydrogenase, lycopene elongase and lycopene cyclase. According to Giani et al. [110], the total number of genes encoding the same enzyme is variable with genera. It could be due to the recent acquisition of those genes through horizontal gene transfer and subsequent recombination events. According to the found carotenogenic enzymes in the studied strains, a carotenoid pathway was proposed from acetyl CoA to bacterioruberin (Fig. 4).

From the above, we can deduce the following facts.

- Comparative and pangenome analyses between the five studied archaeal strains and the well-studied halophilic archaea *Halobacterium* NRC-1 highlighted a notable difference. Many unique genes coding for hypothetical proteins were detected in our strain genomes revealing an environmental adaptation;
- Metabolic pathway analysis revealed similarities and differences between our strains and *Halobacterium* NRC-1. *Halobacterium* NRC-1, contrary to our studied strains, was demonstrated to be able to metabolize glucose despite the presence of a complete semi-phosphorylative Entner-Doudoroff pathway. Compared to *Halobacterium salinarum* NRC-1, the studied strains showed their capabilities to biosynthesize threonine, glycine, methionine and tyrosine amino acids;
- Like other studies, genomic analysis showed a diversity of energetic metabolism (from aerobic respiration to fermentation and anaerobic respiration) and metabolic genes related to sulfate reduction and phosphate solubilization.

## 4 Conclusions

Through genome sequence analysis of the five archaeal strains, this study showed the occurrence of genes encoding enzymes involved in key metabolic processes, including glycolysis/gluconeogenesis, tricarboxylic acid cycle, respiratory chain, pentose phosphate pathway and amino acids metabolism. These strains are predicted to use a variety of metabolic pathways to adapt to high salinity and heavy metal contamination. The genome harbours numerous genes identified and involved in the biosynthesis of vitamins, such as cobalamin, folate, biotin, pantothenate, riboflavin, thiamine, menaquinone, nicotinate and nicotinamide. The presence of these vitamin biosynthetic pathways underscores the biotechnological potential of these studied strains, presenting a natural alternative for industrial vitamin production. The genome analysis also identified several genes involved in ammonium assimilation, phosphate solubilization, chemotaxis, cell motility as well as the production of important plant growth regulator compounds such as indole acetic acid, siderophores and phenazine. Some of these compounds can be used as an eco-friendly biofertilizer, an alternative to chemical fertilizers, to promote plant growth and counteract salt stress effects. Finally, genes related to carotenogenesis have also been identified revealing that the analyzed strains can produce natural compounds which are highly marketed due to the current worldwide trend in which attempts are made to replace the use of chemically synthesized carotenoids with the use of pigments of natural origin.

This study will serve to better understand the metabolism potential of halophilic archaea and to facilitate their manipulation and optimization for novel applications in industrial biotechnology. Nonetheless, further laboratory research is still indispensable to fully elucidate the in-silico predicted metabolic pathways by proteomic and transcriptomic approaches to obtain the real expressed genes.

## CRediT authorship contribution statement

**Houda Baati:** Writing – original draft, Software, Methodology, Investigation, Formal analysis, Data curation. **Mariem Siala:** Writing – review & editing, Methodology. **Souad Benali:** Formal analysis. **Chafai Azri:** Writing – review & editing, Supervision, Conceptualization. **Christopher Dunlap:** Writing – review & editing, Software, Methodology, Formal analysis, Data curation. **Rosa María Martínez-Espinosa:** Writing – review & editing, Supervision. **Mohamed Trigui:** Writing – review & editing, Project administration, Conceptualization.

## Ethical approval

This study does not contain any studies with human or animal participants performed by any authors.

## Data and code availability statement

Data will be made available on request.

## Funding

This research was funded by University of Alicante: PROMETEO/2021/055/Generalirat Valenciana VIGROB-309.

## Declaration of competing interest

The authors declare the following financial interests/personal relationships which may be considered as potential competing interests: Rosa martinez reports financial support was provided by 10.13039/100009092University of Alicante. Rosa martinez reports a relationship with 10.13039/100009092University of Alicante that includes: funding grants. Rosa martinez has patent licensed to PROMETEO/2021/055/Generalirat Valenciana VIGROB-309/University of Alicante. nothing If there are other authors, they declare that they have no known competing financial interests or personal relationships that could have appeared to influence the work reported in this paper.
