# Supplementary material for: Elucidating metabolic pathways through genomic analysis in highly heavy metal-resistant Halobacterium salinarum strains
Source: Heliyon. 2024 Nov 30;10(23):e40822. doi: 10.1016/j.heliyon.2024.e40822 (PMC11665356; doi:10.1016/j.heliyon.2024.e40822)
Supplement: Multimedia component 1 [file mmc1.doc]

**Table S1**

| **Parameter** | **Water samples** | **Sediment samples** |
| --- | --- | --- |
| Salt content (g/l) | 38-300 | 22.26- 8.31 |
| Metals  Pb  Zn  Cu  Ni  Cd | (mg/l)  0.002-0.128  0.065-1,57  0.006-0.064  *  0.002-0.034 | (mg/kg dry weight)  18.5-230.8  27.2-577.1  12.8-101.9  19.2-163.2  5.2-38.9 |

*Not determined

**Table S3**

| **Strain** | | | | | |
| --- | --- | --- | --- | --- | --- |
| **Genes names** | **AS1** | **AS2** | **AS8** | **AS11** | **AS19** |
| **Anaerobic growth on dimethyl sulfoxide (DMSO)**  - Anaerobic dimethyl sulfoxide reductase chain A (EC 1.8.5.3), molybdopterin-binding domain @ Trimethylamine-N-oxide reductase (EC 1.6.6.9)  - Anaerobic dimethyl sulfoxide reductase chain B (EC 1.8.5.3), iron-sulfur binding subunit @ Respiratory trimethylamine-N-oxide reductase, iron-sulfur subunit  - Anaerobic dimethyl sulfoxide reductase chain C (EC 1.8.5.3), anchor subunit @ Respiratory trimethylamine-N-oxide reductase, membrane subunit  - Anaerobic dimethyl sulfoxide reductase chaperone *DmsD*  **Anaerobic growth on arginine**  - Arginine deiminase (EC 3.5.3.6)  - Ornithine carbamoyltransferase (EC 2.1.3.3)  - Carbamate kinase (EC 2.7.2.2)  **Anaerobic growth on Sulfate**  - Thiosulfate sulfurtransferase, rhodanese (EC 2.8.1.1)  - Sulfite oxidase homolog  - Conserved hypothetical protein probably involved in assimilatory sulfate reduction | 1  1  1  1  1  1  1  2  1  1 | 1  1  1  1  1  1  1  2  1  1 | 1  1  1  1  1  1  1  2  1  1 | 1  1  1  1  1  1  1  2  1  1 | 1  1  1  1  1  1  1  2  1  1 |

**Table S4**

| **Strain** | | | | | |
| --- | --- | --- | --- | --- | --- |
| **Genes names** | **AS1** | **AS2** | **AS8** | **AS11** | **AS19** |
| **Pentose phosphate pathway and ribose production**  - 6-phosphogluconate dehydrogenase, decarboxylating (EC 1.1.1.44)  - Translation initiation factor 2B (ribose-1,5-bisphosphate isomerase homologue)  - Deoxyribose-phosphate aldolase (EC 4.1.2.4)  - Ribose-phosphate pyrophosphokinase (EC 2.7.6.1)  - Ribokinase (EC 2.7.1.15)  ***Purine metabolism***  - Nucleoside diphosphate kinase (EC 2.7.4.6)  - Adenylate kinase (EC 2.7.4.3)  - Adenine phosphoribosyltransferase (EC 2.4.2.7)  - Inosine-5'-monophosphate dehydrogenase (EC 1.1.1.205)  - Adenylosuccinate synthetase (EC 6.3.4.4)  - GMP synthase [glutamine-hydrolyzing] (EC 6.3.5.2)  - Adenylosuccinate lyase (EC 4.3.2.2) @ SAICAR lyase (EC 4.3.2.2)  - 5’-nucleotidase SurE (EC 3.1.3.5)  - Xanthine-guanine phosphoribosyltransferase (EC 2.4.2.22)  - IMP cyclohydrolase (EC 3.5.4.10)  - Phosphoribosylglycinamide formyltransferase (EC 2.1.2.2) / Phosphoribosylaminoimidazolecarboxamide formyltransferase (EC 2.1.2.3)  - Phosphoribosylaminoimidazole-succinocarboxamide synthase (EC 6.3.2.6)  - Phosphoribosylformylglycinamidine synthase, synthetase subunit (EC 6.3.5.3)  - Amidophosphoribosyltransferase (EC 2.4.2.14)  - N5-carboxyaminoimidazole ribonucleotide synthase (EC 6.3.4.18)  - N5-carboxyaminoimidazole ribonucleotide mutase (EC 5.4.99.18)  - Adenylate cyclase (EC 4.6.1.1)  - Bis(5'-nucleosyl)-tetraphosphatase (asymmetrical) (EC 3.6.1.17)  - Ribonucleotide reductase of class II (coenzyme B12-dependent) (EC 1.17.4.1)  - Phosphoribosylformimino-5-aminoimidazole carboxamide ribotide isomerase (EC 5.3.1.16)  - Aminodeoxychorismate lyase (EC 4.1.3.38)  - Anthranilate synthase, aminase component (EC 4.1.3.27)  - Isochorismate synthase (EC 5.4.4.2)  - Para-aminobenzoate synthase, amidotransferase component (EC 2.6.1.85)  ***Pyrimidine metabolism***  - Deoxycytidine triphosphate deaminase (EC 3.5.4.13)  - Uracil phosphoribosyltransferase (EC 2.4.2.9)  - Thymidylate kinase (EC 2.7.4.9)  - CTP synthase (EC 6.3.4.2)  - Cytidylate kinase (EC 2.7.4.25)  - Cytidine deaminase (EC 3.5.4.5)  - Uridine phosphorylase (EC 2.4.2.3)  - Thymidine kinase (EC 2.7.1.21)  - Uridine kinase (EC 2.7.1.48)  - Aspartate carbamoyltransferase (EC 2.1.3.2)  - Dihydroorotate dehydrogenase (quinone) (EC 1.3.5.2)  - Nucleoside diphosphate kinase (EC 2.7.4.6)  - Uridylate kinase (EC 2.7.4.22)  - Ribonucleotide reductase of class II (coenzyme B12-dependent) (EC 1.17.4.1)  - Deoxyuridine 5'-triphosphate nucleotidohydrolase (EC 3.6.1.23)  - Thymidylate synthase ThyX (EC 2.1.1.148)  - Bis(5'-nucleosyl)-tetraphosphatase (asymmetrical) (EC 3.6.1.17)  **-** 5'-nucleotidase (EC 3.1.3.5) | 2  1  1  1  1  1  1  2  2  2  2  1  2  1  2  1  1  1  1  2  1  1  1  1  2  1  1  1  1  1  1  1  2  1  1  2  1  1  1  1  1  1  1  1  1  1  1 | 1  1  1  1  1  1  1  1  2  2  2  1  2  1  1  1  1  1  1  1  1  1  1  1  1  1  1  1  1  1  1  1  1  1  1  2  1  1  1  1  1  1  1  1  1  1  1 | 1  1  1  1  1  1  1  1  1  2  2  1  2  1  1  1  1  1  1  1  1  1  1  1  1  1  1  1  1  1  1  1  1  1  1  2  1  1  1  1  1  1  1  1  1  1  1 | 1  1  1  1  1  1  1  1  1  1  2  1  2  1  1  1  1  1  1  1  1  1  1  1  1  1  1  1  1  1  1  1  1  1  1  2  1  1  1  1  1  1  1  1  1  1  1 | 1  1  1  1  1  1  1  1  1  1  2  1  2  1  1  1  1  1  1  1  1  1  1  1  1  1  1  1  1  1  1  1  1  1  1  2  1  1  1  1  1  1  1  1  1  1  1 |

**Table S5**

| **Strain** | | | | | |
| --- | --- | --- | --- | --- | --- |
| **Genes names** | **AS1** | **AS2** | **AS8** | **AS11** | **AS19** |
| **Amino acid synthesis**  **Aspartate / Asparagine / Glutamate / Glutamine /Arginine**  - Aspartate aminotransferase (EC 2.6.1.1)  - Glutamine synthetase type I (EC 6.3.1.2)  - NADP-specific glutamate dehydrogenase (EC 1.4.1.4)  - Asparagine synthetase  - L-aspartate oxidase (EC 1.4.3.16)  - Argininosuccinate synthase (EC 6.3.4.5)  - Argininosuccinate lyase (EC 4.3.2.1)  - Adenylosuccinate lyase (EC 4.3.2.2) @ SAICAR lyase (EC 4.3.2.2)  - Glutamine--fructose-6-phosphate aminotransferase [isomerizing] (EC 2.6.1.16)  - Amidophosphoribosyltransferase (EC 2.4.2.14)  - Carbamoyl-phosphate synthase small chain (EC 6.3.5.5)  **Lysine / Proline**  **-** Ornithine cyclodeaminase (EC 4.3.1.12)  - Proline dehydrogenase (EC 1.5.5.2)  **Alanine / Glycine / Serine / Threonine / Valine / Leucine / Isoleucine**  - Threonine dehydratase, catabolic (EC 4.3.1.19) @ L-serine dehydratase, (PLP)-dependent (EC 4.3.1.17)  - D-3-phosphoglycerate dehydrogenase (EC 1.1.1.95)  - Serine hydroxymethyltransferase (EC 2.1.2.1)  - L-allo-threonine aldolase (EC 2.1.2.1)  - Glycine cleavage system P2 protein (EC 1.4.4.2)  - Aminomethyltransferase (glycine cleavage system T protein) (EC 2.1.2.10)  - Homoserine kinase (EC 2.7.1.39)  - Threonine synthase (EC 4.2.3.1)  **-** Aspartokinase (EC 2.7.2.4)  - Aspartate-semialdehyde dehydrogenase (EC 1.2.1.11)  - 2,3-bisphosphoglycerate-independent phosphoglycerate mutase (EC 5.4.2.12)  - Valine-pyruvate aminotransferase (EC 2.6.1.66)  **Cysteine / Methionine**  - Branched-chain amino acid aminotransferase (EC 2.6.1.42)  - 5-methyltetrahydropteroyltriglutamate-homocysteine methyltransferase (EC 2.1.1.14)  - Cysteine synthase (EC 2.5.1.47)  - S-adenosylmethionine synthetase, archaeal (EC 2.5.1.6)  - Glutamate-cysteine ligase archaeal (EC 6.3.2.2)  - O-acetylhomoserine sulfhydrylase (EC 2.5.1.49)  - Homoserine O-acetyltransferase (EC 2.3.1.31)  - Serine acetyltransferase (EC 2.3.1.30)  - Cystathionine gamma-synthase (EC 2.5.1.48)  - Cystathionine beta-synthase (EC 4.2.1.22)  - Cystathionine gamma-lyase (EC 4.4.1.1)  - Aspartate-semialdehyde dehydrogenase (EC 1.2.1.11)  - S-adenosylhomocysteine deaminase (EC 3.5.4.28)  - DNA-cytosine methyltransferase (EC 2.1.1.37)  **Histidine**  - Histidinol-phosphatase (EC 3.1.3.15)  - Imidazoleglycerol-phosphate dehydratase (EC 4.2.1.19)  - Histidinol dehydrogenase (EC 1.1.1.23)  - Histidinol-phosphate aminotransferase (EC 2.6.1.9)  - Phosphoribosyl-ATP pyrophosphatase (EC 3.6.1.31)  - Phosphoribosyl-AMP cyclohydrolase (EC 3.5.4.19)  - Phosphoribosylformimino-5-aminoimidazole carboxamide ribotide isomerase (EC 5.3.1.16)  - Formiminoglutamase (EC 3.5.3.8)  - Imidazolonepropionase (EC 3.5.2.7)  - ATP phosphoribosyltransferase (EC 2.4.2.17)  - Urocanate hydratase (EC 4.2.1.49)  **Phenylalanine** / **Tyrosine** / **Tryptophan**  - Tryptophan synthase α and ß chain (EC 4.2.1.20)  - 2-amino-3,7-dideoxy-D-threo-hept-6-ulosonate synthase (EC 2.2.1.10)  - 3-dehydroquinate synthase II (EC 1.4.1.24)  - 3-dehydroquinate dehydratase I (EC 4.2.1.10)  - Chorismate synthase (EC 4.2.3.5)  - Shikimate kinase II (EC 2.7.1.71)  - Shikimate 5-dehydrogenase I alpha (EC 1.1.1.25)  - 3-phosphoshikimate 1-carboxyvinyltransferase (EC 2.5.1.19)  - L-tyrosine decarboxylase (EC 4.1.1.25)  - Prephenate and/or arogenate dehydrogenase (unknown specificity) (EC 1.3.1.12)  - Chorismate mutase I (EC 5.4.99.5)  - Prephenate dehydratase (EC 4.2.1.51)  - Biosynthetic Aromatic amino acid aminotransferase beta (EC 2.6.1.57) @ Histidinol-phosphate aminotransferase (EC 2.6.1.9)  - Aminodeoxychorismate lyase (EC 4.1.3.38)  - Isochorismate synthase (EC 5.4.4.2)  **Amino acid degradation**  - Methylaspartate mutase, E and S subunits (EC 5.4.99.1)  - Methylaspartate ammonia-lyase (EC 4.3.1.2)  - Pyruvoyl-dependent arginine decarboxylase (EC 4.1.1.19)  - Agmatinase (EC 3.5.3.11)  - Kynureninase (EC 3.7.1.3) homolog | 2  1  2  1  2  1  1  1  2  1  1  3  1  1  3  1  1  1  1  1  1  2  1  2  1  1  2  1  1  1  1  1  2  1  1  2  1  2  3  1  1  2  1  1  1  2  1  2  1  1  2  1  1  1  1  1  1  1  1  1  2  1  1  1  1  2  1  1  1  1 | 1  3  3  1  1  1  1  1  1  1  1  2  1  1  2  1  1  1  1  1  1  1  1  1  1  1  2  1  1  1  1  1  1  1  1  1  1  2  4  1  1  1  1  1  1  1  1  1  1  1  2  1  1  1  1  1  1  1  1  2  1  1  1  1  1  2  1  1  1  1 | 1  1  3  1  1  1  1  1  1  1  1  2  1  1  2  1  1  1  1  1  1  1  1  1  1  1  2  1  1  1  1  1  1  1  1  1  1  2  1  1  1  1  1  1  1  1  1  1  1  1  2  1  1  1  1  1  1  1  1  1  1  1  1  1  1  2  1  1  1  1 | 1  1  3  1  1  1  1  1  1  1  1  2  1  1  2  1  1  1  1  2  1  1  1  1  1  1  2  1  1  1  1  1  1  2  1  1  1  2  1  1  1  1  1  1  1  1  1  1  1  1  2  1  1  1  1  1  1  1  1  1  1  1  1  1  1  2  1  1  1  1 | 1  1  3  1  1  1  1  1  1  1  1  2  1  1  2  1  1  1  1  2  1  1  1  1  1  1  2  1  1  1  1  1  1  2  1  1  1  2  1  1  1  1  1  1  1  1  1  1  1  1  2  1  1  1  1  1  1  1  1  1  1  1  1  1  1  2  1  1  1  1 |

**Table S6**

| **Strain** | | | | | |
| --- | --- | --- | --- | --- | --- |
| **Name of the enzymes** | **AS1** | **AS2** | **AS8** | **AS11** | **AS19** |
| **Mevalonate pathway**  - Acetyl-CoA acetyltransferase (EC 2.3.1.9)  - Hydroxymethylglutaryl-CoA synthase (EC 2.3.3.10)  - Hydroxymethylglutaryl-CoA reductase (EC 1.1.1.34)  - Mevalonate kinase (EC 2.7.1.36)  - Phosphomevalonate decarboxylase (EC 4.1.1.99)  - Isopentenyl phosphate kinase (EC 2.7.4.26)  **Isoprene biosynthesis**  - Isopentenyl-diphosphate delta-isomerase (EC 5.3.3.2)  - Geranylgeranyl diphosphate synthase (EC 2.5.1.29) (*CrtE*)  - Undecaprenyl diphosphate synthase (EC 2.5.1.31)  **Carotenoids biosynthesis**  - Phytoene synthase (EC 2.5.1.32) (*CrtB*)  - Phytoene dehydrogenase (EC 1.14.99.-)  - Phytoene desaturase (*CrtI*) (EC 1.14.99.-)  **-** Lycopene elongase (*LyeJ*) (EC 2.5.1.-)  - Lycopene cyclase (CrtY) | 2  1  1  1  2  1  3  2  1  2  3  1  1  1 | 2  1  1  1  1  1  2  2  1  2  2  1  1  1 | 2  1  1  1  1  1  1  2  1  2  2  1  1  1 | 2  1  1  1  1  1  1  2  1  2  2  1  1  1 | 2  1  1  1  1  1  1  2  1  2  3  1  1  1 |
